# 

**Published:** 1854-06

**Authors:** 


☞ Owing to the scarcity of paper and the breaking down of the mill that usually supplies us, this number of the Journal is delayed much beyond the usual time of publication.

